# Effects of copper nanoparticle exposure on host defense in a murine pulmonary infection model

**DOI:** 10.1186/1743-8977-8-29

**Published:** 2011-09-24

**Authors:** Jong Sung Kim, Andrea Adamcakova-Dodd, Patrick T O'Shaughnessy, Vicki H Grassian, Peter S Thorne

**Affiliations:** 1 Interdisciplinary Graduate Program in Human Toxicology, University of Iowa, Iowa City, IA 52242, USA; 2 Department of Occupational and Environmental Health, University of Iowa, Iowa City, IA 52242, USA; 3 Department of Chemistry, University of Iowa, Iowa City, IA 52242, USA

**Keywords:** Copper, nanoparticles, inhalation, instillation, bacterial clearance, murine, pulmonary infection, *Klebsiella pneumoniae*

## Abstract

**Background:**

Human exposure to nanoparticles (NPs) and environmental bacteria can occur simultaneously. NPs induce inflammatory responses and oxidative stress but may also have immune-suppressive effects, impairing macrophage function and altering epithelial barrier functions. The purpose of this study was to assess the potential pulmonary effects of inhalation and instillation exposure to copper (Cu) NPs using a model of lung inflammation and host defense.

**Methods:**

We used *Klebsiella pneumoniae *(*K.p.*) in a murine lung infection model to determine if pulmonary bacterial clearance is enhanced or impaired by Cu NP exposure. Two different exposure modes were tested: sub-acute inhalation (4 hr/day, 5 d/week for 2 weeks, 3.5 mg/m^3^) and intratracheal instillation (24 hr post-exposure, 3, 35, and 100 μg/mouse). Pulmonary responses were evaluated by lung histopathology plus measurement of differential cell counts, total protein, lactate dehydrogenase (LDH) activity, and inflammatory cytokines in bronchoalveolar lavage (BAL) fluid.

**Results:**

Cu NP exposure induced inflammatory responses with increased recruitment of total cells and neutrophils to the lungs as well as increased total protein and LDH activity in BAL fluid. Both inhalation and instillation exposure to Cu NPs significantly decreased the pulmonary clearance of *K.p.*-exposed mice measured 24 hr after bacterial infection following Cu NP exposure versus sham-exposed mice also challenged with *K.p *(1.4 × 10^5 ^bacteria/mouse).

**Conclusions:**

Cu NP exposure impaired host defense against bacterial lung infections and induced a dose-dependent decrease in bacterial clearance in which even our lowest dose demonstrated significantly lower clearance than observed in sham-exposed mice. Thus, exposure to Cu NPs may increase the risk of pulmonary infection.

## Background

Due to the expanding use of nanoparticles (NPs) and rapid growth in nanotechnology, the potential for human exposure has increased tremendously. The U.S. National Science Foundation estimates that nanotechnology will have a $1 trillion impact on the global market and will employ over 7 million workers by 2015 [1]. Among different types of nanomaterials, metal and metal oxide NPs have already found numerous consumer applications and are major components of manufactured nanomaterials [2]. This large number of exposed workers means that even a small increase in risk can lead to significant morbidity from nanomaterial exposure.

NPs show novel physicochemical properties that emerge at the nanoscale and affect their interaction with biological systems and processes [3,4]. It is likely that exposures to NPs will occur in conjunction with microbial agents. Innate host defense mediated through neutrophils is critical to control bacterial clearance in the host [5]. Although it is well established that neutrophils are in the front line of host defense against bacterial infection, the exact role of neutrophils in host defense against bacterial infection (e.g. *M. tuberculosis*) is still not fully elucidated [6]. It is of primary importance for host defense to maintain neutrophil concentrations at appropriate physiologic levels [7]. Inflammation may be induced through exposure to inhaled NPs [8,9] by triggering complex interactions between the drivers and response elements of inflammation and oxidative stress [10].

Copper (Cu) NPs are widely synthesized and used as metal catalysts, heat transfer fluids in machine tools, semiconductors, and even in antimicrobial preparations [11,12]. Since Cu NPs are one of the primary engineered NPs in industrial applications, concerns over the emission of engineered NPs into the environment and consequent potential adverse effects on human health have increased.

Our group has studied the toxicity of a variety of nanomaterials in mice [8,9,13]. We found Cu NPs induced stronger inflammatory responses than oxides of iron (Fe), titanium (Ti), or silver (Ag) with increased recruitment of total cells and neutrophils to the lungs as well as increased total protein and lactate dehydrogenase (LDH) activity in bronchoalveolar lavage (BAL) fluid. Cytotoxicity and DNA damage were also reported in A549 type II lung epithelial cells from all metal oxide particles tested (CuO, TiO_2_, ZnO, Fe_2_O_3_, Fe_3_O_4_) at 40 and 80 μg/mL [2]. Overall, CuO NPs have been reported to be among the more toxic nanomaterials in mammals as indicated by inflammation in mice exposed sub-acutely [9]. Therefore, it is especially important to assess interactions with Cu-based NPs and host defense against microbial infections.

Both inhalation and instillation exposure systems have recognized limitations [14]. Several studies have reported that the inhalation route was more effective in assessing an inflammatory response, oxidative stress, collagen deposition and fibrosis [8-10,13,15]. However, the actual dose delivered to the lungs can be more clearly defined with instillation exposure. There are also concerns with use of intratracheal instillation exposure. This is a non-physiological and invasive particle delivery technique that deposits particles in a bolus and produces an uneven distribution. Furthermore, instilled particles bypass the upper respiratory tract that plays an important role in adverse effects of inhaled particles [14,16]. Several studies have been conducted in an attempt to bridge two different exposure modes for assessment of NP toxicity, namely inhalation and instillation exposures [8,17,18]. These investigators found lower inflammatory responses in animals exposed by inhalation as opposed to instillation exposure, however, there is generally more uncertainty in the assessment of retained dose in inhalation studies.

Both Cu and Ag NPs are known to have anti-microbial activity *in vitro *[19]. However, NPs may inhibit microbial clearance by inducing excessive neutrophil-mediated inflammation [10,20]. To address this question, we first established a murine pulmonary infection model of *Klebsiella pneumoniae (K.p.) *following NP exposure. We chose *K.p.*, an organism known to cause infection and pneumonia in mammals. *K.p. *also affects persons with impaired immune systems such as chronic lung disease [21]. We designed a sub-acute inhalation and acute intratracheal instillation NP study with mice challenged with *K.p. *to determine if pulmonary clearance is enhanced or impaired by Cu NP exposure as compared to sham-exposure. We also characterized pulmonary responses and the body burden of Cu measured in the tissues after NP exposure.

## Materials and methods

### Source and Characterization of NPs

Cu NP powders (partially passivated with oxygen by the manufacturer) were purchased and used as received from the manufacturer (Nanostructured and Amorphous Materials, Inc, Houston, TX, USA). The Cu NPs were characterized using X-ray diffraction (XRD), transmission electron microscopy (TEM), X-ray photoelectron spectroscopy (XPS) and BET techniques as described previously [9]. The Cu NPs show a core/shell morphology with a metallic Cu core and an oxidized shell consisting of Cu_2_O (Cuprite) and CuO (tenorite), with CuO on the surface of the particles. A compilation of some of the properties measured for the commercially manufactured NPs after purchasing are listed in Table 1.

**Table 1:** Summary of physicochemical characterization data of Cu NPs [9] and experimental design of inhalation and instillation studies of pulmonary bacterial clearance

Physicochemical property	Value
Primary particle size (TEM)	12 ± 1 nm
Crystalline phases (XRD)	Cu, Cu_2_O, CuO*
Surface functionality (XPS)	O, O-H and H_2_O
Surface area (BET)	12 ± 0.2 m^2^/g

Pulmonary bacterial clearance study	

Sub-acute inhalation (NP aerosol concentration)	3.5 ± 0.4 mg/m^3^; 4 hr/d, 5 d/wk, 2 wk
Aerosol size distribution**	200 nm (1.4)
Acute instillation (NP exposure concentration)	3, 35 and 100 μg/mouse; 24 hr post-exposure
Bacterial infection concentration	1.4 ± 0.1 × 10^5 ^CFUs/mouse
Necropsy post-infection of bacteria	24 hr

*Cu NPs show a core/shell type morphology with a metallic Cu core and an oxidized shell consisting of a Cu_2_O inner shell and a CuO outer surface. See Ref [27] for further details. The oxidized shell increases in thickness relative to the core upon aging of the NPs in the ambient environment, i.e. upon long term exposure to water vapor and molecular oxygen.

**Geometric mean, nm, (geometric standard deviation) in whole-body exposure chamber

### Animals

Male 6 week old (22-25 g) C57Bl/6 mice (The Jackson Laboratory, Bar Harbor, ME) were utilized in both the inhalation and instillation studies. Protocols were approved by the Institutional Animal Care and Use Committee and complied with the NIH Guide for the Care and Use of Laboratory Animals. Mice were held in quarantine for 12 days prior to use in AAALAC-accredited vivarium in polypropylene, fiber-covered cages in HEPA-filtered Thoren caging units (Hazelton, PA). They were supplied with food (sterile Teklad 5% stock diet, Harlan, Madison, WI) and water *ad libidum *and maintained on a 12-hr light-dark cycle.

**Figure 1:**
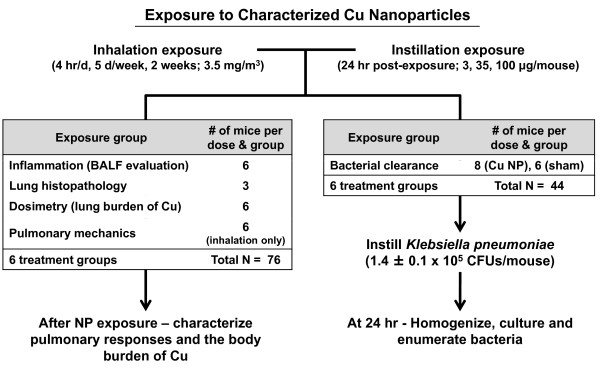
**Schematic of the pulmonary bacterial clearance model**. We established a murine pulmonary infection model of *Klebsiella pneumoniae (K.p.) *to determine if pulmonary bacterial clearance is impaired by Cu nanoparticle exposure using two different exposure modes, inhalation and intratracheal instillation. Following both sub-acute inhalation (4 hr/d, 10 d; 3.5 mg/m^3^; 32 μg cumulative dose) and intratracheal instillation (24 hr post-exposure; 3, 35, 100 μg/mouse), mice were intratracheally challenged with *K.p. *bacteria at a dose of 1.4 ± 0.1 × 10^5 ^CFUs/mouse.

The experimental design and number of mice used per dose and group is shown in Figure 1. Briefly, pulmonary responses and the body burden of Cu measured in the tissues and BAL fluids were characterized after Cu NP exposure or sham exposure (filtered lab air for inhalation and optima water for instillation study). We also evaluated lung bacterial clearance following exposures. In the sub-acute inhalation study, additional mice were exposed to Cu NP or filtered lab air for assessment of pulmonary mechanics.

### NP Exposure, Aerosol Generation and Characterization

For the inhalation study, a dynamic whole-body exposure system was used to expose mice to Cu NP aerosols as previously described [8]. Briefly, mice were placed within sectioned, open-wire cages that were positioned in a whole-body, custom-fabricated aluminum exposure chamber [22]. A suspension of Cu powder in water (Optima grade, Fisher Scientific, Pittsburgh, PA) was nebulized to generate a Cu NP aerosol. A 1 mg Cu NP/mL suspension was first ultra-sonicated with a high frequency probe set at 30% of the maximum amplitude (20 kHz, model 550, Fisher Scientific, Pittsburgh, PA) for 20 min to minimize the degree of agglomeration. The suspension was then placed in a 6-jet Collison nebulizer (BGI Inc., Waltham, MA) supplied with filtered, pressurized air. The droplets were dried by passing them through a 110°C brass drying column and the dry Cu NPs entered a tube containing a 20 mCi ^63^Ni source to remove static charge prior to entering the chamber.

We measured the time-integrated mass concentration of the aerosol in the chamber by gravimetric analysis of 47-mm glass microfiber filters (Whatman, Middlesex, United Kingdom) in line with the 24 L/min exhaust air flow. Sample grids for TEM were placed inside the exposure chamber to measure agglomerate sizes of the NPs. The size distribution of the aerosol in the whole-body exposure chamber during inhalation exposures was measured using a scanning mobility particle sizer (SMPS, model 3080 electrostatic classifier with model 3081 differential mobility analyzer and model 3785 condensation particle counter, TSI Inc., Shoreview, MN) that measured diameters in the range of 7.4 to 289 nm according to the manufacturer's instructions. Geometric mean (GM) and geometric standard deviation (GSD) of aerosol sizes in individual exposures were determined from the SMPS measurements.

### Sub-acute Inhalation Exposure

In sub-acute studies, mice were exposed 4 hr/day, 5 d/wk for 2 weeks and necropsied immediately after exposure. The average concentration of Cu NPs was 3.5 ± 0.4 mg/m^3^, very close to our previous sub-acute exposure concentration of the same NPs (3.7 mg/m^3^) [9]. Sham-exposed controls breathed filtered laboratory air in identical exposure chambers in an adjacent laboratory. The deposited dose was estimated assuming minute volume of 25 mL/min and a deposition fraction of 15% in the tracheobronchial and pulmonary region [23,24] yielding an estimated NP dose of 32 μg Cu NP/mouse. This estimation assumes there is no clearance *via *the mucociliary escalator to the trachea or gastrointestinal tract and no translocation of NPs to other organs.

### Intratracheal Instillation Exposure

Three concentrations of Cu NPs (3, 35, and 100 μg/mouse) were used for intratracheal instillation exposures. These were prepared by suspending particles in Optima water to minimize contaminants found in unprocessed water. This suspension was ultrasonicated as described above and then vortexed immediately before dosing to minimize aggregation of particles. Mice were anesthetized by isoflurane inhalation prior to intratracheal instillation of 50 μL of Cu NP suspensions. Animals were positioned on an inclined restraining stand and a very flexible FEP (Fluorinated ethylene propylene) polymer catheter (24G 3/4", Smiths Medical International Ltd., UK) affixed to a 1-mL syringe was then inserted into the mouth and placed between the vocal cords and into the lumen of the trachea. Illumination of the trachea was provided by a directed fiber optic light source. Mice instilled with 50 μL Optima water alone were used as negative controls. Experimental groups were necropsied 24 hr after the instillation (24 hr post-exposure).

### Pulmonary Bacterial Infection and Clearance Model

*K.p. *was obtained from the American Tissue Culture Collection (ATCC 43816, Rockville, MD). The bacteria were inoculated into 50 mL of tryptic soy broth (TSB) in 250 mL flasks for 18 hr (stationary phase) with shaking (120 rpm) in a gyratory water bath shaker at 37°C (New Brunswick Scientific Co., Edison, NJ). The bacterial suspension was diluted in TSB to obtain an optical density (OD_660_) of 0.4 by measuring in a Spectra Max microplate reader (Molecular Device, Sunnyvale, CA); 200 μL of this solution was added to 50 mL of TSB for 3 hr to reach mid-log phase of growth (OD_660 _~ 0.4, corresponding to ~2 × 10^8 ^colony-forming units (CFUs/mL), where bacteria are most virulent.

Immediately following the 10^th ^sub-acute inhalation or the intratracheal instillation exposure of Cu NPs, mice were anesthetized by isoflurane inhalation and then intratracheally instilled with a bacterial inoculum containing 1.4 ± 0.1 × 10^5 ^CFUs *K.p. *per mouse incorporated into and mixed with amorphous agar particles (molten 4% Noble Agar, BD, Franklin Lakes, NJ) in a 50 μL volume as described previously [25]. Twenty-four hr after *K.p. *inoculation, mice were euthanized by isoflurane inhalation and the lungs were removed and whole lung tissues were suspended in 1 mL of sterile saline, homogenized and cultured on tryptic soy agar (TSA). The number of bacteria remaining in the lungs was counted after an overnight incubation at 37°C. The concentrations of instilled *K.p. *were checked by spread plating the inocula on TSA. The dose and duration of bacterial inoculation were based on preliminary studies showing that this bacterial dose did not cause mortality and could be cleared effectively in control mice.

### Evaluation of Bronchoalveolar Lavage (BAL) Fluid

Six mice per group were euthanized by isoflurane inhalation and each lung was lavaged 3 times (total volume, 3 mL) with 0.9% sterile sodium chloride solution (Baxter, Deerfield, IL). BAL fluid was collected, processed and the cell pellet was used for enumeration of total and differential cell counts. The lavage supernatants were analyzed for total protein levels using a Bradford protein assay (Bio-Rad Laboratories, Inc., Hercules, CA), LDH activity measured with a commercial assay (Roche Diagnostics, Penzberg, Germany) and cytokines were quantified by multiplexed fluorescent bead-based immunoassays (Bio-Rad Laboratories, Inc., Hercules, CA). Seven inflammatory cytokines and chemokines were chosen based on our previous study [9] and included interleukin [IL]-6, IL-12(p40), tumor necrosis factor [TNF]-α, granulocyte macrophage colony stimulating factor [GM-CSF], keratinocyte-derived cytokine [KC], monocyte chemotactic protein [MCP]-1, and macrophage inflammatory protein [MIP]-1α.

### Lung Histopathology

Lungs from 3 mice per group that were not lavaged were fixed in 10% formaldehyde-phosphate-buffered saline solution *via *the canulated trachea. The tissues were subsequently paraffin-embedded, sectioned at 5 μm, and stained with hematoxylin and eosin (H & E) as previously described [26]. Tissue sections were evaluated by light microscopy in four categories each employing a five point scale to elucidate abnormalities of the parenchymal architecture (bronchioles, alveoli, pleura, and vasculature); abnormal inflammatory infiltrates; presence or absence of acute lung injury; and presence or absence of fibrosis.

### Determination of Cu Concentration in the Tissues and BAL Fluids

To determine the total amount of Cu in the lung, brain, heart, kidney, and splenic tissues, tissues were removed from Cu NP-exposed and sham-exposed mice, frozen (-80°C) and lyophilized for 16 hr at 1.3 × 10^-1 ^mBar and -50°C in a freeze dryer (Labconco Corp., Kansas City, MO) and then weighed. Mixtures of high purity concentrated nitric acid and hydrochloric acid (Fisher Optima^® ^grade) were used to digest the tissues with a HotBlock™ digestion system (Environmental Express, Mt. Pleasant, SC) at 95-98°C. Digestates were diluted to 10 mL with Optima water and Cu analysis was performed using an inductively coupled plasma-mass spectrometer (ICP-MS, X Series, Thermo Scientific). To determine dissolved Cu ion concentration in the lung tissues after NP exposure, the BAL fluid was centrifuged at 44,912 *g *for 30 min to separate NPs and aggregates that were not dissolved. Cu concentration in the particle-free BAL supernatants was measured by ICP-MS.

### Pulmonary Mechanics Measurements

Pulmonary mechanics were measured using the forced oscillation technique. Mice were anesthetized with 90 mg/kg of pentobarbital sodium (Ovation Pharmaceuticals, Inc., Deerfield, IL) by intraperitoneal injection. Tracheotomy was performed using a tracheal cannula with luer adapter (1.3 mm, length 20 mm, Harvard Apparatus, MA) and each mouse was connected to a computer-controlled small animal ventilator (flexiVent, SCIREQ, Montreal, QC, Canada). The mice were ventilated at 150 breaths/min with a tidal volume of 10 mL/kg and positive end-expiratory pressure of 2-3 cm H_2_O.

To measure airway hyperactivity, mice were challenged with increasing concentrations of methacholine aerosols (1, 3, and 10 mg/mL), generated with an in-line nebulizer (10 sec) directly through the canulated trachea. Response to each methacholine dose was measured every 30 sec for 5 min. Only data with a coefficient of determination greater than 0.95 were included in the final analysis. The lungs were inflated to total lung capacity after each methacholine dose. A sinusoidal (single-frequency) forced oscillation waveform ("snapshot perturbation") maneuver was performed to measure resistance (R), compliance (C), and elastance (E) of the whole respiratory system (airways, lungs and chest wall). A broadband (multi-frequency) forced oscillation waveform was used to produce measurements of airway resistance (Rn), tissue damping (G) and tissue elastance (H). All these parameters were calculated using flexiVent software (version 5). Responses were expressed by computation of the area under the curve (AUC) of each parameter measured versus time for each 5 min methacholine dose monitoring period.

### Statistical Analyses

Data from mice exposed to Cu NPs were compared to sham-exposed mice using two-sided *t *tests for equal or unequal variances and one-way analyses of variance (ANOVA) was done for comparison between groups in the instillation study (SAS Ver. 9.2, SAS, Inc., Cary, NC). Results from pulmonary mechanics measurements were tested for outliers using the stem-and-leaf and normal probability plots produced by the univariate procedure (SAS). We calculated the AUC for each parameter (e.g., R, C and E) versus time plot for all methacholine doses. For the inhalation study, the means and AUCs of pulmonary mechanics parameters for the Cu-exposed group were compared to sham-exposed mice using ANOVA for repeated measures. A *p*-value less than 0.05 was considered statistically significant. All data are expressed as mean ± standard error (SE) unless otherwise noted.

## Results

### Particle Characterization

We used manufactured Cu NPs that had been previously well-characterized [9]. Evaluation by TEM of NPs revealed an average primary particle size of 12 ± 1 nm, smaller than the manufacturer's stated particle size of 25 nm. XRD of the NPs showed the presence of three phases, metallic Cu core, Cu_2_O and CuO. The thickness of these oxidized layers depends on both the preparation of the NPs and exposure to air as NPs age under atmospheric conditions [27]. The surface area of Cu NPs was 12 ± 0.2 m^2^/g by a multi-point BET analysis. Measurement of airborne NPs showed a mobility diameter of 200 nm and GSD of 1.4 (Table 1).

### Sub-acute Inhalation Study

**Figure 2:**
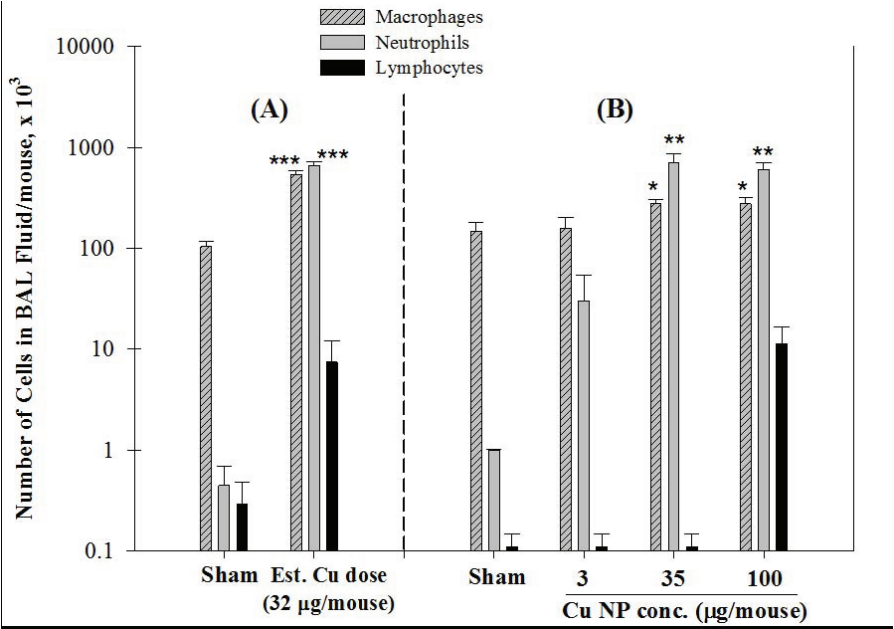
**Differential cell analysis of BAL fluid in animals exposed to Cu nanoparticles (NPs)**. Number of cells in BAL fluid from mice after sub-acute inhalation (A) and intratracheal instillation (B) exposure of Cu NPs. Data are expressed as mean ± SE; *p < 0.05, **p < 0.01, ***p < 0.001 significantly different from sham-exposed mice; Macrophages (*p *< 0.05) and Neutrophils (*p *< 0.001) from instillation exposure were analyzed by one-way ANOVA test of a trend for comparison between NP exposure groups and controls.

As shown in Table 2, total cell counts of BAL fluid in mice after sub-acute inhalation exposure to Cu NPs (1,207 ± 106 × 10^3^cells/mouse) were significantly increased from sham-exposed mice (105 ± 14 × 10^3^, *p *< 0.001). We found significant differences in total protein levels (544 ± 38 μg/mL, *p *< 0.001) and activity of LDH (336 ± 29 U/L, 340% of control) from BAL fluid in Cu-exposed mice compared with sham-exposed mice (Table 2). Macrophages and neutrophils in BAL fluid were significantly increased in mice exposed to Cu particles and necropsied immediately after exposure (Figure 2, 54% of neutrophils influx, *p *< 0.001).

**Table 2:** Total number of cells, total protein and activity of LDH in BAL fluid after sub-acute inhalation (4 hr/d, 10 d; 3.5 mg/m^3^) and intratracheal instillation (24 hr post-exposure; 3, 35, 100 μg) to Cu NPs

Animal Group	**Total number of cells/mouse (× 10**^**3 **^**± SE)**	Total protein (μg/mL ± SE)	LDH Activity (U/L ± SE)
Inhalation study			

Sham exposure	105 ± 14	127 ± 4	100 ± 6
Cu NP	1,210 ± 106***	544 ± 38***	336 ± 29***

Instillation study			

Sham exposure	149 ± 32	107 ± 7	163 ± 17
Cu NP Low, 3 μg/mouse	186 ± 70	122 ± 17	193 ± 21
Cu NP Medium, 35 μg/mouse	977 ± 156**	160 ± 13*	236 ± 21*
Cu NP High, 100 μg/mouse	882 ± 121**	219 ± 37*	341 ± 39**
^*†*^*p *by one-way ANOVA	*p *< 0.001	*p *< 0.01	*p *< 0.001

Data are expressed as mean ± SE; *p < 0.05, **p < 0.01, ***p < 0.001 significantly different from sham-exposed mice.

^*†*^*p-*value from one-way ANOVA test of a trend for comparison between NP exposure groups and controls.

**Figure 3:**
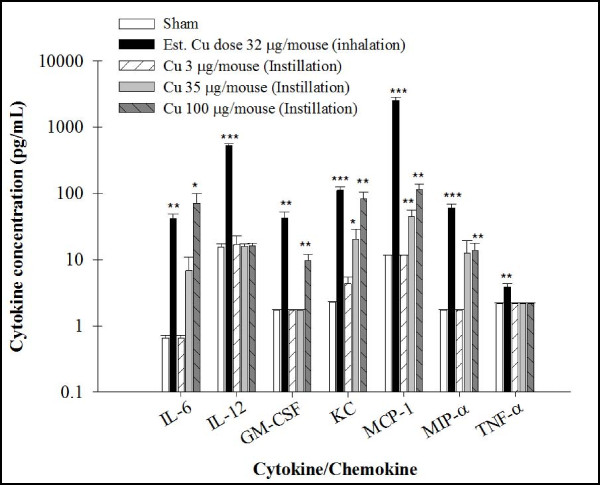
**Cytokine/chemokines analysis of BAL fluid in animals exposed to Cu nanoparticles (NPs)**. Cytokine/chemokine concentrations in BAL fluid from mice after sub-acute inhalation and intratracheal instillation exposure of Cu NPs. Data are expressed as mean ± SE; *p < 0.05, **p < 0.01, ***p < 0.001 significantly different from sham-exposed mice; IL-6 (*p *< 0.05), GM-CSF, KC, MCP-1 and MIP-α (*p *< 0.001) from instillation exposure were analyzed by one-way ANOVA test of a trend for comparison between NP exposure groups and controls.

In comparison with sham-exposed mice, all seven inflammatory cytokines/chemokines in lavage fluid were significantly increased in the Cu-exposed group as shown in Figure 3: IL-6 (*p *< 0.01), IL-12 (*p *< 0.001), GM-CSF (*p *< 0.01), KC (*p *< 0.001), MCP-1 (*p *< 0.001), MIP-1α (*p *< 0.001), and TNF-α (*p *< 0.01).

### Intratracheal Instillation Study

Mice exposed *via *intratracheal instillation demonstrated a dose-dependent increase in total cells recovered from BAL fluid (Table 2). Total cell counts were significantly elevated in mice exposed to medium (*p *< 0.05) and high concentrations (*p *< 0.001) of Cu NP when compared to control mice. Total protein and LDH activity were significantly elevated with exposure to the medium (both *p *< 0.05, 150% of control) and high concentration (*p *< 0.05, 200% of control; *p *< 0.01, 210% of control, respectively) of Cu particles, when compared to controls. Mice exposed to the high concentration had significantly higher levels of total protein and LDH activity than those exposed to the low concentration of particles (*p *< 0.05, both).

The number of neutrophils per mouse in BAL fluid was significantly increased with exposure to the medium and high concentration of NPs (*p *< 0.05 and *p *< 0.01, respectively), when compared to controls (Figure 2). We also found significant differences in the number of alveolar macrophages between the medium and high concentrations of Cu NP-exposed mice compared with controls (*p *< 0.05, both).

In comparison with sham-exposed mice, cytokine/chemokines levels were significantly increased at the high exposure level for IL-6 (*p *< 0.05), GM-CSF (*p *< 0.01), KC (*p *< 0.001), MCP-1 (*p *< 0.01), and MIP-α (Figure 3). The concentration of KC and MCP-1 was significantly increased in BAL fluid at the medium dose of instilled Cu NPs (*p *< 0.05 and *p *< 0.01, respectively).

### Lung Histopathologic Evaluation

**Figure 4:**
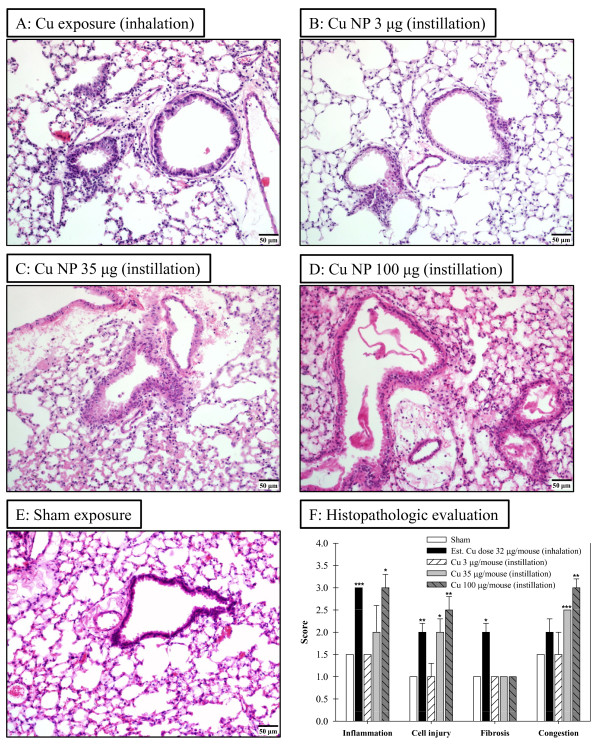
**Representative micrographs and quantitative histopathologic evaluations of lung tissues from animals exposed to Cu nanoparticles (NPs)**. Micrographs of lung sections stained with H&E from mice exposed to Cu NPs by sub-acute inhalation (A) or intratracheally instilled with a low (3 μg, B), medium (35 μg, C), or high (100 μg, D) dose or sham-exposed (E). Histopathologic evaluation was performed by light microscopy in four categories (inflammation, cell injury, fibrosis, and congestion) each employing a five point scale (F). Data are expressed as mean ± SE; *p < 0.05, **p < 0.01, ***p < 0.001 significantly different from sham-exposed mice.

Representative micrographs and quantitative histopathologic evaluations of lung tissues are shown in Figure 4. Vascular congestion, perivascular inflammation (mainly mononuclear and neutrophilic) and apoptotic bodies were seen in airspaces and near sites of inflammation in all Cu-exposed mice (Figures 4A-D). Extremely mild foci of perivascular inflammation (mostly mononuclear/histiocytic) were found in sham-exposed mice (Figure 4E). In mice exposed sub-acutely (Figure 4A), we found moderate to severe inflammation (score 3, mostly lymphoid and lesser neutrophilic, *p *< 0.001) in perivasculitis, perialveolitis and alveolar areas with some luminal sloughed cells, lacking overt fibrosis (score 2, *p *< 0.05). Pulmonary epithelia had foci of mucus change. Vascular congestion (score 2) and apoptotic bodies were seen in air spaces and near areas of inflammation.

Mice exposed to instilled Cu NPs showed dose-dependent inflammation, cell injury, and congestion (Figure 4B-D, and 4F). The low exposure groups exhibited rare foci of scant inflammation in perivascular and peribronchiolar areas (Figure 4B). Lung tissues from these mice and sham-exposed mice were evaluated as normal (inflammation score 1.5 and cell injury score 1). Mild pigment-laden macrophages near sites of congestion (score 2.5, *p *< 0.001) were seen and airway epithelium had rare sloughing of apparently apoptotic cells (score 2, *p *< 0.05) in the medium concentration of Cu NP-instilled mice (Figure 4C). Moderate to severe vascular congestion (score 3, *p *< 0.01) and uncommon foci of mild peribronchiolar neutrophils were found in the high concentration group (Figure 4D). Severe congestion with foci of alveolar edema developing and with neutrophils and perivasculitis and alveolitis with severe increase of inflammation were also seen in this group.

### Pulmonary Bacterial Clearance

**Figure 5:**
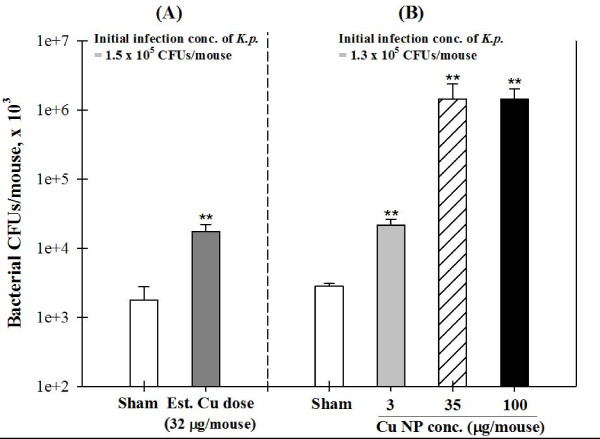
**Assessment of pulmonary bacterial clearance of *Klebsiella pneumoniae (K.p.) *in mice following inhalation and instillation of Cu nanoparticles (NPs)**. Immediately after the final sub-acute inhalation exposure (A) or the instillation exposure (B) of Cu NPs, mice were intratracheally administered 1.4 ± 0.1 × 10^5 ^CFUs/mouse *K.p. *Lungs were homogenized 24 hr after bacterial inoculation and the number of CFUs was counted after culturing at 37°C. Data are expressed as mean ± SE; **p < 0.01 significantly different from sham-exposed mice.

To determine the effects of Cu NP exposure on host defense against bacterial infection, we examined 24 hr lung clearance of instilled *K.p. *in mice that had been exposed sub-acutely to Cu NPs. Despite the purported anti-bacterial activity of Cu, Cu NP exposure significantly decreased the bacterial clearance as compared to sham-exposed mice also challenged with *K.p. *(Figure 5, *p *< 0.01). As shown in Figure 5, bacterial clearance from the lungs of mice exposed by instillation was also significantly decreased (*p *< 0.05) with increasing doses of Cu NPs (3 μg, 8-fold; 35 μg, 520-fold; 100 μg, 510-fold). Pulmonary bacterial clearance was significantly blunted in the medium and high concentration Cu-exposed mice compared to the low concentration (*p *< 0.01). However, this effect reached a plateau and we did not observe significant differences between the medium and high concentration groups (Figure 5) both of which demonstrated bacterial growth beyond what was introduced.

### Dosimetry of Cu NPs in the Tissues and BAL Fluids

**Figure 6:**
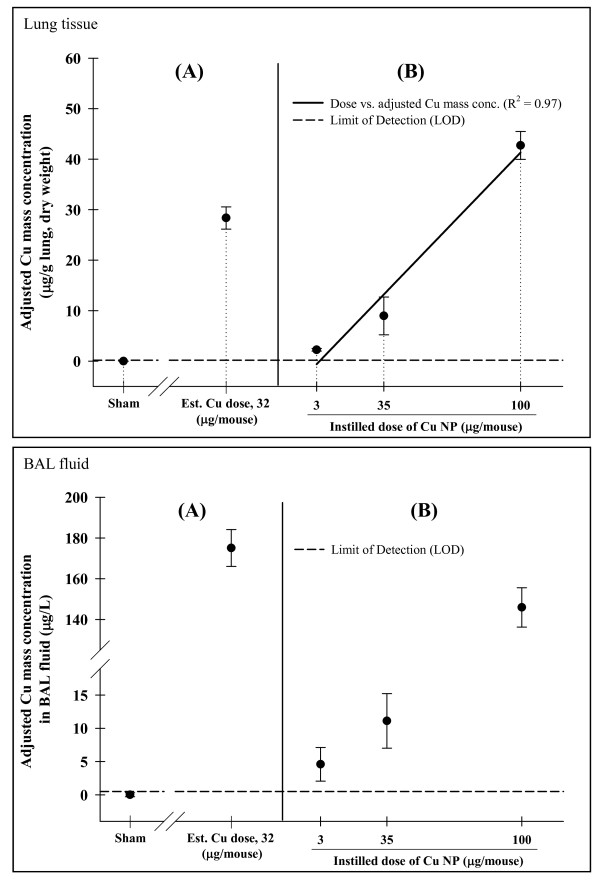
**Cu retained in the lung tissues and BAL fluids in inhalation- and instillation-exposed mice**. Total amounts of Cu in the lung tissues (upper) and BAL fluids (lower) from mice immediately after sub-acute inhalation (A) and instillation (B) of Cu nanoparticles (NPs). The lung burdens of Cu NP in Cu-exposed mice were adjusted for the level of Cu in sham-exposed mice.

Cu retained in the lung tissues and BAL fluids in inhalation- and instillation-exposed mice were measured by ICP-MS (Figure 6). The mass of Cu (adjusted for the Cu in sham-exposed mice) without or with lung lavages following sub-acute inhalation exposure was 39 ± 3 and 28 ± 2 μg/g lung (dry wt) (*p *< 0.001), respectively. The concentration of Cu ions in the particle-free BAL supernatants was 175 ± 9 μg/L in mice necropsied immediately after inhalation exposure (Figure 6B). The Cu lung burdens of instillation-exposed mice after lung lavages 24 hr post-exposure increased in a dose-dependent manner from 2 to 9 to 43 μg/g lung (Figure 6A). These amounts represent the burdens in the lungs alone (i.e. not the nose, nasopharynx or trachea). The concentration of Cu ions in BAL fluids from instilled Cu-exposed mice increased dose-dependently from 5 to 11 to 146 μg/L. The dry weight of lungs and volume of BAL fluids are shown in additional file 1. The amount of Cu measured in the brain, heart, kidney, and splenic tissues from inhalation-exposed mice was not significantly elevated compared to sham-exposed mice (additional file 2).

### Pulmonary Mechanics from Inhalation Study

**Figure 7:**
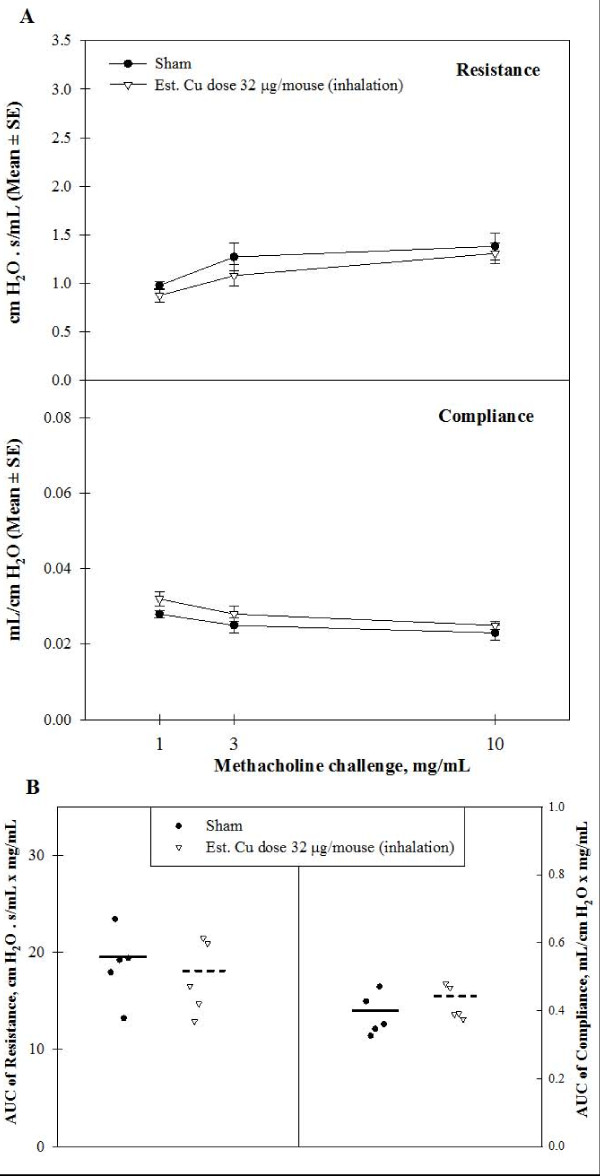
**Measurement of pulmonary mechanics in mice sub-acutely exposed to Cu nanoparticles**. Pulmonary mechanics measurements expressed as resistance and compliance in sham- and Cu-exposed mice after increasing concentration of methacholine (1, 3, 10 mg/mL). The mean and SE of resistance and compliance versus methacholine concentrations (A). The individual and mean area under the curve (AUC) versus all increasing methacholine concentrations (B).

Pulmonary mechanics parameters such as resistance, elastance, compliance, airway resistance, tissue damping and tissue elasticity were assessed in mice challenged with increasing doses of methacholine aerosol. Comparison of means of AUC between controls and Cu-exposed mice using ANOVA for repeated measures showed no significant differences (Figure 7). Our data indicate that inhalation exposure to Cu NPs at this dose did not result in clinically significant remodeling of the airway or airway hyperreactivity of the mice.

## Discussion

Impairment of bacterial host defense can arise from reduced macrophage or neutrophil function or a reduction in the activity of innate anti-microbial peptides. Immediately following inhalation exposure, Cu-exposed mice showed increased inflammation compared to sham-exposed mice indicated by the number of total cells (12-fold) and neutrophils (1,460-fold), and concentrations of cytokines: IL-6 (62-fold), IL-12 (35-fold), GM-CSF (25-fold), KC (49-fold), MCP-1 (218-fold), MIP-α (35-fold), and TNF-α (2-fold). Increased levels of total protein (4-fold) and LDH activity (3-fold) in lung lavage fluid (Table 2) provided evidence of cytotoxicity. Inflammatory responses were induced in a dose-dependent fashion as indicated by levels of total protein, and LDH activity in BAL fluids following instillation exposure of Cu NPs. Mice exposed to the medium or high dose of Cu NPs had significantly greater numbers of neutrophils (23-fold and 20-fold, respectively) than mice exposed to the low concentration.

Recent studies have demonstrated the key role of cytokine/chemokines and inflammatory cells to pulmonary inflammation and bacterial infections [5,28,29]. It has been reported in these studies that the neutrophil chemoattractant KC is essential for recruiting neutrophils and regulating other chemokines such as MIP and LIX (lipopolysaccharide-induced CXC chemokine) to provide innate immune responses against bacterial infection from the lungs in mice. KC resulted in enhanced accumulation of monocytes/macrophages in addition to neutrophils at the site of infection as well as higher lung levels of IL-12. TNF-α is a critical early cytokine required for neutrophil recruitment. MCP-1 also can amplify neutrophil and macrophage recruitment in the lung. These elevations were consistent with increased numbers of BAL macrophages and neutrophils and histopathologic evaluation of lung tissues of Cu-exposed mice (perivasculitis and alveolitis). Pulmonary functions of mice exposed to Cu by inhalation were not impaired; this is consistent with our histopathology findings, where no edema or fibrosis was found. In mice with fibrosis we would expect an increase in resistance and a decrease in compliance in comparison to controls [30].

Cu NPs impaired pulmonary bacterial clearance. Mice exposed acutely or sub-acutely NPs had significantly more viable bacteria in the lungs than sham-exposed mice. We did not observe significant differences in bacterial clearance between mice exposed to the medium and high concentration of instilled Cu NPs and there was also no difference in lung neutrophils in these groups. Interestingly, even though the amount of Cu found in the lungs after instillation exposure of Cu NPs was representative of the exposure doses, and increased as expected in a dose-dependent fashion, the recruitment of neutrophils and macrophages and the degree of pulmonary clearance plateaued at the medium dose of instilled Cu NPs. The roles of neutrophil influx on host defense against bacterial infection are controversial. An insufficient neutrophil recruitment results in decreased bacterial clearance, whereas an excessive neutrophil influx can lead to severe neutrophil-mediated inflammation and lung injury [5,31] that could cause decreasing bacterial clearance from lungs. Li and colleagues addressed the questions of why host defense against bacterial and fungal infections is compromised when neutrophil concentration falls below a critical threshold value. They reported that a bacterial concentration in excess of a critical threshold concentration of neutrophils, resulted in reduced bacterial killing by neutrophils [7]. Thus, the rate of bacterial clearance appears to depend on the ratio of neutrophils to bacteria.

A recent study reported that carbon nanotube pre-exposure significantly decreased the pulmonary bacterial clearance despite robust inflammatory responses [10]. These authors reported that bacterial clearance might depend more on production of nitric oxide than ROS from the oxidative burst. Our data also demonstrate that exposure to Cu NP *via *inhalation and instillation attenuated lung bacterial clearance even though there was a robust inflammation.

It was previously suggested by our group that the inflammatory response induced by Cu NPs in mice was potentially associated with the solubility of Cu NPs *in vivo *[9]. The amount of Cu found in the unlavaged lungs after inhalation exposure was 39 ± 3 μg/g lung or 8% of the 520 μg/g lung that we estimate was delivered to the mice. Cu remaining in the lung tissues after lavage was 28 ± 2 μg/g lung. Dissolved free Cu ions or Cu NPs taken up by inflammatory cells on the airway surface may have been lavaged from the alveolar and bronchial airspaces. Cu measured in the lungs of instilled mice 24 hr post-exposure increased in a dose-dependent manner. Moreover, increasing doses of instilled Cu NP produced both increasing concentrations of Cu measured in BAL fluids (5, 11 and 146 μg/L) after centrifugation at 44,912 *g *for 30 min and increasing concentrations of Cu measured in digested lungs after lavage (2, 9 and 43 μg/g lung). Sharma et al. have demonstrated that centrifuging at 5,600 *g *for 30 min effectively separates NPs down to 16 nm size [32]. Thus, these dosimetry data provide evidence of Cu dissolution occurring in the lungs. To test the hypothesis if an inflammatory response seen after exposure to Cu NPs is due to Cu ions or particles, we performed limited experiments with metal NPs and their nitrate compounds (completely dissolved). The metal nitrates including Cu nitrate induced more severe inflammatory responses than the metal NPs at the same molar concentration (data not shown). We expect more dissolved Cu ions at the high dose of Cu NPs, thus, increasing the inflammatory responses.

## Conclusions

Our study indicates that Cu NP exposure induced an impairment in host defense against bacteria in both inhalation and instillation exposure studies even though there was an upregulation of pro-inflammatory cytokines and recruitment of neutrophils to the lungs. Thus, Cu NP exposure may lead to increased risk of pulmonary infection by impairing host defense against bacteria.

## Abbreviations

Ag: silver; ANOVA: analyses of variance; AUC: area under the curve; CFU: colony-forming unit; Cu: copper; FEP: fluorinated ethylene propylene; GM: geometric mean; GM-CSF: granulocyte macrophage colony stimulating factor; GSD: geometric standard deviation; H & E: hematoxylin and eosin; ICP-MS: inductively coupled plasma-mass spectrometer; IL: interleukin; KC: keratinocyte-derived cytokine; *Kp*: *Klebsiella pneumoniae; *LDH: lactate dehydrogenase; LIX: lipopolysaccharide-induced CXC chemokines; MCP: monocyte chemotactic protein; MIP: macrophage inflammatory protein; NP: nanoparticle; ROS: reactive oxygen species; SE: standard error; SMPS: scanning mobility particle sizer; TEM: transmission electron microscopy; TNF: tumor necrosis factor; TSA: tryptic soy agar; TSB: tryptic soy broth; XPS: X-ray photoelectron spectroscopy; XRD: X-ray diffraction; ZnO: zinc oxide

## Competing interests

Vicki H. Grassian is a consultant and a member of the scientific advisory board of Vive Nano (Toronto, Canada) and holds stock in Nanoscale Corp. (Manhattan, Kansas).

## Authors' contributions

JSK participated in the study design, conducted the animal exposure studies, the biological assays, data analysis, and drafted the manuscript; AAD participated in the study design, animal studies including pulmonary mechanic measurements and data interpretation; PTO coordinated NP generation for inhalation exposures and aerosol characterization; VHG contributed to the analyses of NP characterization data and integrated study design; PST designed animal studies and coordinated the biological assays and data analysis and authored the final manuscript. All authors read and approved the final manuscript.

## Supplementary Material

Additional file 1 **Total amounts of Cu in the lung tissues and BAL fluids from mice immediately after inhalation (4 hr/d, 10 d; 3.5 mg/m^3^) and instillation (24 hr-post exposure; 3, 35, 100 μg/mouse) of Cu NPs. The lung burdens of Cu NP in Cu-exposed mice were adjusted for the level of Cu in sham-exposed mice**. The mass and concentration of Cu in lung tissues and BAL fluids from Cu NP-exposed mice and the dry weight of lungs and volume of BAL fluids. Click here for file

Additional file 2 **Total amounts of Cu in the tissues from mice enrolled in the sub-acute exposure study taken immediately after the final inhalation exposure (4 hr/d, 10 d; 3.5 mg/m^3^)**. The amount of Cu measured in the brain, heart, kidney, and splenic tissues from inhalation-exposed mice and the limit of detection (LOD) for ICP-MS analysis. Click here for file
