# Supplementary material for: Effects of copper nanoparticle exposure on host defense in a murine pulmonary infection model
Source: Part Fibre Toxicol. 2011 Sep 24;8:29. doi: 10.1186/1743-8977-8-29 (PMC3193802; doi:10.1186/1743-8977-8-29)
Supplement: Additional file 1 — Total amounts of Cu in the lung tissues and BAL fluids from mice immediately after inhalation (4 hr/d, 10 d; 3.5 mg/m3) and instillation (24 hr-post exposure; 3, 35, 100 μg/mouse) of Cu NPs. The lung burdens of Cu NP in Cu-exposed mice were adjusted for the level of Cu in sham-exposed mice. The mass and concentration of Cu in lung tissues and BAL fluids from Cu NP-exposed mice and the dry weight of lungs and volume of BAL fluids. [file 1743-8977-8-29-S1.PDF]

**Additional file 1 (with Figure 6).** Total amounts of Cu in the lung tissues and BAL fluids from mice immediately after inhalation (4 hr/d, 10 d; 3.5 mg/m$^3$) and instillation (24 hr-post exposure; 3, 35, 100 μg/mouse) of Cu NPs. The lung burdens of Cu NP in Cu-exposed mice were adjusted for the level of Cu in sham-exposed mice. Data are expressed as mean ± SE.

| Exposure group | Lung | | | BAL fluid | | |
|---|---|---|---|---|---|---|
| | Cu concentration (μg/g) | Lung dry weight (mg) | Cu mass (μg) | Cu concentration (μg/L) | Volume (mL) | Cu mass (μg) |
| Inhalation | 28.4 ± 2.2 | 55.2 ± 2.5 | 1.6 ± 0.1 | 175.1 ± 9.0 | 3.4 | 0.60 ± 0.03 |
| Instillation | | | | | | |
| 3 μg/mouse | 2.3 ± 0.3 | 25.5 ± 1.2 | 0.1 ± 0.0 | 4.6 ± 2.5 | 2.9 | 0.01 ± 0.01 |
| 35 μg/mouse | 9.0 ± 3.7 | 28.0 ± 1.5 | 0.3 ± 0.1 | 11.1 ± 4.1 | 3.3 | 0.04 ± 0.01 |
| 100 μg/mouse | 42.7 ± 2.8 | 34.6 ± 1.7 | 1.5 ± 0.1 | 145.9 ± 9.7 | 3.0 | 0.45 ± 0.03 |
