# Supplementary material for: Effects of copper nanoparticle exposure on host defense in a murine pulmonary infection model
Source: Part Fibre Toxicol. 2011 Sep 24;8:29. doi: 10.1186/1743-8977-8-29 (PMC3193802; doi:10.1186/1743-8977-8-29)
Supplement: Additional file 2 — Total amounts of Cu in the tissues from mice enrolled in the sub-acute exposure study taken immediately after the final inhalation exposure (4 hr/d, 10 d; 3.5 mg/m3). The amount of Cu measured in the brain, heart, kidney, and splenic tissues from inhalation-exposed mice and the limit of detection (LOD) for ICP-MS analysis. [file 1743-8977-8-29-S2.PDF]

Additional file 2 (with Figure 6). Total amounts of Cu in the tissues from mice enrolled in the sub-acute exposure study taken immediately after the final inhalation exposure (4 hr/d, 10 d; 3.5 mg/m^3).

Tissue	Cu mass concentration (μg/g tissue, dry weight)		Limit of Detection (LOD)	
	Sham exposure	Cu exposure	mg/L	μg/g tissue
Brain	11.3 ± 2.0	14.5 ± 2.1	0.06	5.8
Heart	19.3 ± 1.0	21.1 ± 1.4	0.02	6.7
Kidney	10.7 ± 0.6	12.3 ± 0.8	0.06	5.3
Spleen	15.8 ± 5.7	13.3 ± 3.9	0.02	10.7
